# Demand management processes to improve access to cognitive-behavioral therapies for anxiety disorders: a grounded theory study

**DOI:** 10.3389/frhs.2023.1266987

**Published:** 2024-01-11

**Authors:** Jean-Daniel Carrier, Frances Gallagher, Alain Vanasse, Pasquale Roberge

**Affiliations:** ^1^ PRIMUS Research Group, Department of Family Medicine and Emergency Medicine, Université de Sherbrooke, Sherbrooke, QC, Canada; ^2^ Department of Psychiatry, Université de Sherbrooke, Sherbrooke, QC, Canada; ^3^ School of Nursing, Université de Sherbrooke, Sherbrooke, QC, Canada; ^4^ Centre de Recherche du CHUS, Sherbrooke, QC, Canada

**Keywords:** anxiety disorders, access to health services, demand management, clinical-administrative processes, grounded theory

## Abstract

**Introduction:**

Anxiety disorders are impactful mental health conditions for which evidence-based treatments are available, notably cognitive-behavioral therapies (CBTs). Even when CBTs are available, demand-side factors limit their access, and actors in a position to perform demand management activities lack a framework to identify context-appropriate actions.

**Methods:**

We conducted a constructivist grounded theory study in Quebec, Canada, to model demand management targets to improve access to CBTs for anxiety disorders. We recruited key informants with diverse experiences using purposeful, then theoretical sampling. We analyzed data from 18 semi-directed interviews and 20 documents through an iterative coding process centered around constant comparison.

**Results:**

The resulting model illustrates how actors can target clinical-administrative processes fulfilling the demand management functions of detection, evaluation, preparation, and referral to help patients progress on the path of access to CBTs.

**Discussion:**

Modeling clinical-administrative processes is a promising approach to facilitate leveraging the competency of actors involved in demand management at the local level to benefit public mental health.

## 1 Introduction

Anxiety disorders are prevalent mental health conditions (1) associated with decreased quality of life (2) and potentially significant long-term disability (3). Other consequences of anxiety disorders include increased risks of chronic physical conditions (4), depression with poor outcomes (5, 6), mortality from both natural and unnatural causes (7), and suicide in particular (8, 9). Anxiety disorders disproportionately affect women and individuals younger than 35 (10, 11). However, they are often undetected in men and older adults, notably because of differences in help-seeking attitudes and behaviors (12, 13). Age and gender notwithstanding, some individuals with untreated anxiety disorders avoid health services because of their symptoms (14); in contrast, others seek excessive reassurance, inappropriately use emergency rooms, or undergo superfluous and potentially invasive investigations in search of an explanation for their symptoms (14, 15). Consequently, barriers to accessing treatment for anxiety disorders extend beyond treatment availability.

Even in high-income countries, about a third of individuals with anxiety disorders receive any treatment for their condition, while as little as 15% receive what could be considered an evidence-based treatment (16). Those proportions are halved in middle-income countries (16). Individuals may be more likely to benefit from medications, psychological interventions, or both (15, 17). Medications are supported by a reliable distribution network and may, therefore, be more accessible (18), but most people living with anxiety disorders favor psychological interventions over medications when given a choice (19). Psychological interventions targeting anxiety disorders are generally based on cognitive-behavioral therapy (CBT) principles and techniques, including modalities such as individual psychotherapy delivered by CBT specialists, group psychotherapy, guided internet-based interventions, and self-directed treatment packages (15). While the diversity of evidence-based CBT delivery modalities creates new opportunities to make those treatments more available (20), this is not a sufficient condition to guarantee access to everyone.

In addition to treatment availability, access requires the perception of needs and desire for care, health care seeking, the ability to afford health care utilization, and the ability to engage appropriate services, all of which can be considered from the perspective of either supply- or demand-side actors (21). Adopting this multidimensional perspective, a scoping review on improving access to CBTs for anxiety disorders has found six types of strategies from the thematic analysis of 94 published documents: contributing to the evidence base, identifying CBT delivery modalities to adopt in practice, building capacity for CBT delivery, attuning the process of access to local needs, engaging potential service users, and improving programs and policies (20). This review highlighted that actors who may contribute to improving access are diverse and should be targeted by strategies fitting their situation. In particular, supply-side actors may consider different strategies depending on the systems they belong to, including the delivery system, the support system, or the knowledge synthesis and translation system (22).

Despite previous attempts to distinguish between categories of actors involved in improving access, some might still not recognize their perspectives in the published literature. One example is primary care clinicians, who may not understand their role as belonging to the CBT delivery system when they recommend CBTs for their patients. In that respect, *demand management* would be a more fitting conceptualization of many actors' involvement in access to CBTs for anxiety disorders (23). In particular, actors performing demand management activities address unmet and unidentified needs of CBTs for anxiety disorders in various populations (24), both prompting individuals with needs to seek and access appropriate interventions and ensuring that intervention seekers actually need what they seek (25). Given the existing gaps in the scientific literature concerning those important actors, this study aimed to model demand management targets to improve access to CBT modalities for anxiety disorders that may already be available in an individual's local context.

## 2 Material and methods

### 2.1 Study design

Given this study's objective, we opted for a grounded theory design, which is a methodical approach to inductively generate a theoretical framework from empirical data (26). We conducted this grounded theory study using the constructivist approach conceptualized by Kathy Charmaz (27). Constructivist grounded theory was the best fit for this study's topic, considering that (1) various actors would be expected to differ in their interpretations of the processes involved in improving access to CBTs for anxiety disorders, and (2) contextual factors would have a major influence on the specifics of their actions. The Guideline for Reporting and Evaluating Grounded Theory research studies (28) and Standards for Reporting Qualitative Research (29) guided the writing of this article.

### 2.2 Researchers' perspective

When conducting grounded theory research, the researchers' prior experience and intent cannot be isolated from theory development (27). Consequently, we note that this study was conducted in the context of the first author's Ph.D. (30). Moreover, the first author worked as a consulting psychiatrist in a family medicine practice during this study, placing him in a specialist role in assisting demand management activities for patients with anxiety disorders and other mental health conditions. All participants were provided with this information, and efforts to mitigate the researchers' perspective's impact on data collection and analysis are described in the corresponding sections.

### 2.3 Setting

We conducted this study in Quebec, a Canadian province with a majority French-speaking population totaling approximately 8.6 million (31). In Quebec, mental health-related care has been explicitly legislated under the province's Professional Code since 2012. This framework defines psychotherapy as an intervention restricted to certified psychotherapists who may be psychologists, physicians, or members of some other professions with additional training. However, interventions based on CBT principles are not restricted if they meet definitions explicitly distinguishing them from psychotherapy, such as “psychological education”, “supportive intervention”, or “clinical follow-up” (32).

Most of Quebec's certified psychotherapists practice in the private sector, with services either payable out-of-pocket, covered by private (and generally employment-related) insurance plans, or falling under a short list of public insurance plans (33). Psychological interventions are publicly covered when provided by psychotherapists employed by Quebec's public healthcare establishments network or by physicians (33). Quebec's government is also currently investing in the ability of public sector non-psychotherapist staff to provide lower-intensity interventions based on CBT principles (34). Lastly, depending on the region, nonprofit organizations may provide some interventions based on CBT principles for free or at low cost, although they do not generally employ certified psychotherapists.

### 2.4 Participants and recruitment

Given the breadth of the study's topic, we elected to recruit key informants as participants, i.e., people who would be able and willing to inform us not only from their own experience but from their knowledge of prevailing social currents (35). We sampled participants for qualitative interviews in two steps: purposeful sampling, followed by theoretical sampling. During purposeful sampling (2018–2019, *n* = 8), we sought to maximize the variety of participants' relevant experiences (35) by targeting combinations of the following profiles: clinicians, managers (private, public, or nonprofit sectors), policymakers, and researchers. During theoretical sampling (2019–2020, *n* = 10), we selected new participants to recruit by considering how their perspectives could contribute to developing a better understanding of emerging categories, their properties, and their interrelations (27).

Although this study was intended to have implications for people with anxiety disorders, we did not focus on this population for recruitment. Indeed, this study investigated the actions and processes leading to improving access to CBTs for anxiety disorders from a demand management perspective, and the actors involved in these actions and processes constituted the study population. We identified participants based on the researchers' knowledge and professional networks, using public information on the internet, or on the suggestion of previous participants or potential participants who declined our invitation. We first contacted potential participants through email addresses publicly available or provided by a third party, or using the direct messaging function of professionals-oriented social networks (e.g., researchgate.net, linkedin.com).

### 2.5 Data collection

The first author conducted semi-directive qualitative interviews using an interview guide constructed and validated through consensus by research team members. Although the interview process was flexible, we approached the interview guide's construction in four general sections: participant profile and experiences, views on the current and ideal situations of access to CBTs for anxiety disorders, perceived facilitators and barriers to access, and possible avenues for action. As theorization progressed, we focused interview questions on the conceptual categories emerging from data analysis and adapted the interview guide accordingly. We summarize the interview guide's content throughout the study in Supplementary Appendix S1. Evolving versions of the interview guide are available in French in the first author's Ph.D. thesis (30).

We conducted interviews either at the location of the participant's choice or using the videoconference software Zoom (36, 37). Interviews were recorded using a handheld device or the software's recording function and transcribed integrally by an administrative professional who signed a confidentiality undertaking. The interviewer reviewed every transcript for accuracy while listening to the audio recording. Two co-researchers (PR, FG) each listened to at least three of the purposely sampled interviews and gave the interviewer feedback to improve his performance in subsequent interviews and facilitate the transition to theoretical sampling.

In addition to interviews, we included extant documents either provided or evoked by interview participants, gathered through research team members' experiences and networks, or elicited by internet searches throughout data analysis. In constructivist grounded theory, extant documents are rich data sources for theorizing insofar as the researchers have not influenced their creation and can analyze both their form and content (27, 28).

### 2.6 Data analysis

**Figure 1:**
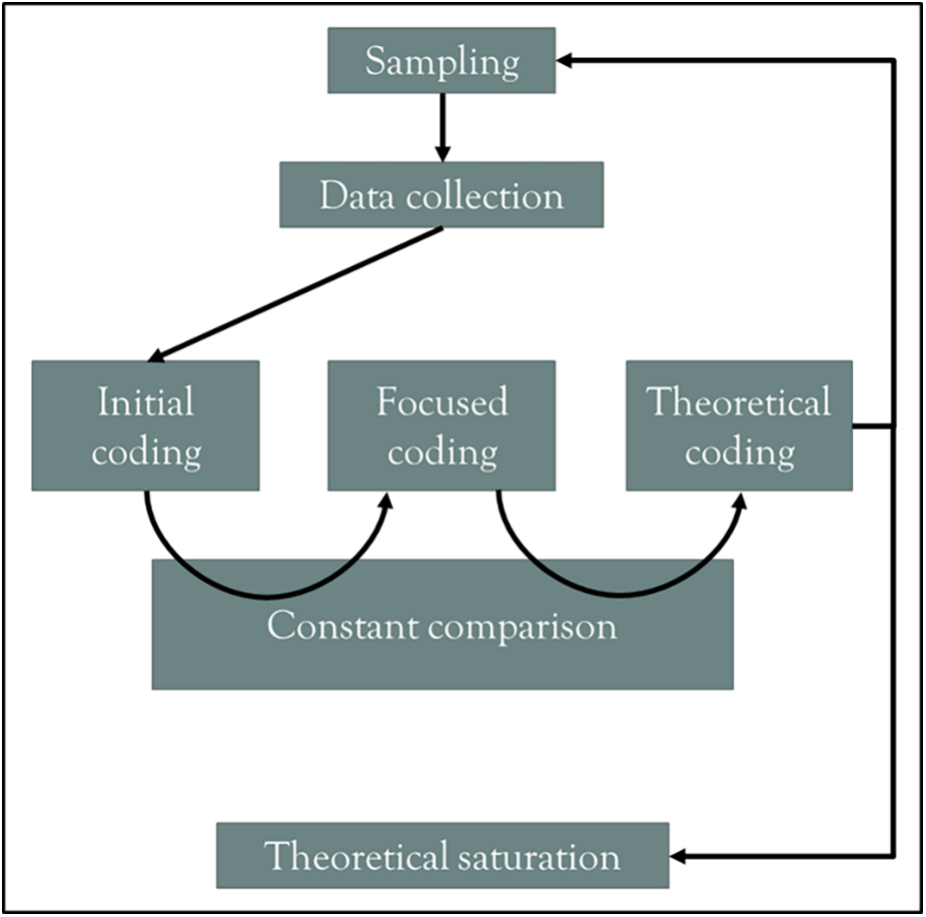
Data analysis process for this grounded theory study.

Interview transcripts and extant documents were uploaded to the NVivo software (38). We performed data analysis following an inductive process in three phases, illustrated in Figure 1: initial coding, focused coding, and theoretical coding.

We performed line-by-line initial coding for all interview transcripts and documents, attributing a descriptive code to each quote directly linked to the research objective (27, 39). Starting from initial codes, during focused coding, we isolated some potential categories that could refer to and operationalize actions improving access to CBTs for anxiety disorders. Examining those categories and their potential interrelationships during theoretical coding, we iteratively constructed a model of potential targets that actors involved in demand management could consider to improve access to CBTs for anxiety disorders. Two co-researchers (PR, FG) co-coded a total of five interviews and gave feedback on the initial and focused codes at crucial moments of data analysis. The coding process spanned almost three years, during which we regularly held team discussions.

The transition between initial, focused, and theoretical coding proceeded by constant comparison aided by systematic memo writing. We compared initial codes and the corresponding quotes when transitioning to focused coding. When transitioning to theoretical codes, we compared aspiring categories and their potential interrelationships between themselves and to previously published frameworks and perspectives. Two frameworks used during theoretical coding substantially influenced the final model's structure: Levesque et al.'s *Conceptual framework of access to health care* (21) and Aiken et al.'s *Organizational behavior classification and modeling framework* (40).

As illustrated in Figure 1, each iteration of theoretical coding led to decisions on the next step in sampling until theoretical saturation was achieved, i.e., reaching the conceptual density necessary to account for each conceptual component of the developing model (27). We used Proctor et al.'s recommendations to help operationalize theoretical saturation in this study, leading to identifying processes involving specific actors, actions, action targets, temporality, and targeted outcomes (41).

### 2.7 Ethical considerations

This study was approved in July 2018 by the CIUSSS de l'Estrie-CHUS research ethics committee in Sherbrooke, Quebec (MP-31-2019-2840). Each participant provided written informed consent before their interview and retained the right to terminate their participation at any point, with the caveat that their contribution to the researchers' theoretical sensitivity and understanding of the subject matter as recorded in memos could not be excluded from the analysis. We edited any quotes presented to minimize the risk of participants' identification, each of whom was assigned a randomly generated two-digit number specifically for this article.

## 3 Results

### 3.1 Data sources

Eighteen key informants from seven of Quebec's health regions participated in qualitative interviews, eleven of which were completed before the COVID-19 pandemic. Table 1 summarizes the participants' profiles. Interviews were conducted in French and had an average duration of 89 min (range 59–113). Interviews tended to span longer during purposeful than theoretical sampling, averaging 99 and 82 min, respectively.

**Table 1:** Interview participants’ profiles.

Characteristics	Number (total = 18)
Gender	
F	10
M	8
Age	
30–39	1
40–49	7
50–59	4
60–69	5
70–79	1
CBT providers (*n* = 9)	
Certified psychotherapist	7
Bachelor-level provider	1
Peer provider	1
Profession (*n* = 14)	
Clinical psychologist^a^	7
Psychiatrist	2
General physician	1
Other (social worker, psychoeducator, career counsellor, criminologist)	4
Policymaking role (*n* = 7)	
Government official	2
Expert consultant	5
Management experience (*n* = 11)	
Public healthcare system	6
Nonprofit organization	4
Insurance provider	1
Research experience (*n* = 7)	
Independent investigator	4
Other	3

^a^ Most participating clinical psychologists reported clinical experience in both public and private sectors.

Table 2 presents the documents included and references them when applicable. Twenty documents were coded for analysis, five of which were provided by participants and cannot be shared to preserve confidentiality (Doc1-Doc5). Eight of the remaining documents were guidelines or policy-related (Doc6, Doc8, Doc10-Doc14, Doc16), four were peer-reviewed journal articles (Doc7, Doc9, Doc16, Doc17), two were an editorial (Doc19) and a transcript from a round table (Doc20) published in a peer-reviewed journal's thematic issue, and one was the annual report of a professional organization (Doc15).

**Table 2:** Documents included and analyzed in this study.

Document number	Reference	Description
Doc1	Provided by participant (P14)	Internal communication from a regional health organization
Doc2	Provided by participant (P14)	Internal guidelines from a regional health organization
Doc3	Provided by participant (P14)	Internal management tool from a regional health organization
Doc4	Provided by participant (P27)	Annual report of a nonprofit organization
Doc5	Provided by participant (P63)	Book therapy recommendations list
Doc6	AMPQ, (42)	Recommendations for organizing one-stop access to mental health services
Doc7	Bradley et al., (43)	Point of view of psychologists and psychotherapists on government-funded psychotherapy (article)
Doc8	CACBT, (44)	Recommendations for CBT training in Canada
Doc9	Grenier et al., (45)	Advocacy for the inclusion of psychologists in primary care teams (article)
Doc10	MSSS, (46)	Guidelines on the organization of frontline mental health services
Doc11	MSSS, (47)	Mental Health Action Plan 2015–2020
Doc12	MSSS, (48)	Provincial strategy to support intersectoral actions
Doc13	MSSS, (34)	Guidelines on the identification and service trajectories for common mental disorders
Doc14	OPQ, (49)	Position statement on the practice of evidence-based psychotherapy
Doc15	OPQ, (50)	Annual report of a professional organization (*Ordre des psychologues du Québec*)
Doc16	Renaud-Gagnon et al., (51)	Deployment guide for primary care adult mental health services
Doc17	Roy, Dagenais, et al., (52)	Literature review on optimizing the mental health services continuum (article)
Doc18	Roy, Pinsonneault, et al., (53)	Implementation evaluation of a model of local consultation in health and social services (article)
Doc19	Vasiliadis et al., (54)	Editorial of a special issue on improving access to psychotherapies in Quebec and Canada
Doc20	Vasiliadis et al., (55)	Results of a round table on improving access to psychotherapies in Quebec and Canada

As all the interview transcripts and most of the documents included were in French, the first author translated the relevant quotes into English.

### 3.2 Design and scope of the model

**Figure 2:**
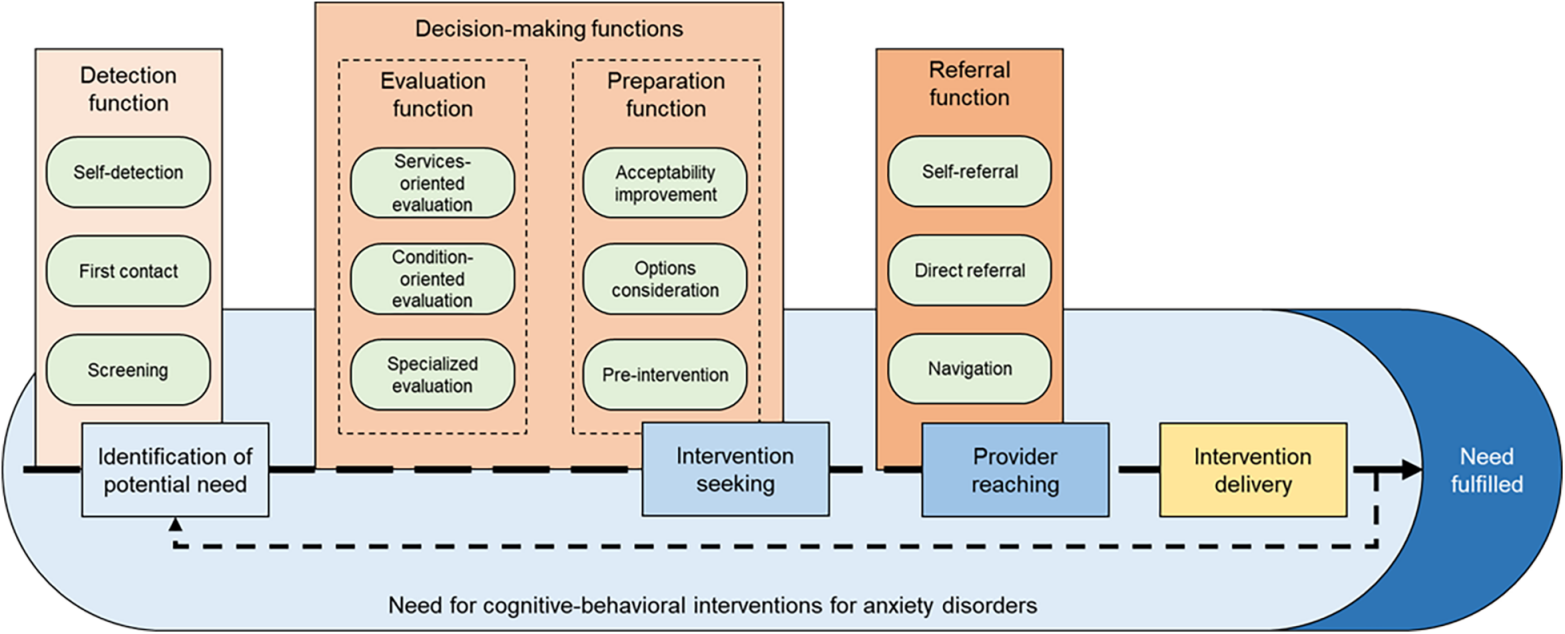
Clinical-administrative processes on patients' access paths to CBTs.

We found that demand management actions aiming to improve access to CBTs can be modeled along the access path of individuals with anxiety disorders who need this treatment, hereafter referred to as patients. As shown in Figure 2 (blue), we conceptualized that the access path involves a series of related outcomes: identification of potential need, intervention seeking, and provider reaching. This path then leads to intervention delivery (yellow), after which persisting needs must be reidentified when applicable, as indicated by the backtracking arrow. The path toward access can be interrupted at any stage, represented by the dashed lines.

Our results indicate that demand management actions fulfill specific clinical-administrative functions (shades of brown) connected to patients' access paths. The adjective “clinical-administrative”, first heard from a participant (P38) to describe their role in case management, reflects the inherent challenges of reconciling patients' and health services' perspectives in demand management. How specific actors may fulfill clinical-administrative functions depends on the nature and context of their interactions with patients, leading to distinct clinical-administrative processes (green) associated with each function.

The conceptual definitions of functions, processes, and how they relate to each other were inspired by Aitken et al.'s *Organizational behavior classification and modeling framework* (40). Consequently, we define a function as a capability of the mental health system associated with an identifiable goal. Comparatively, a process is a sequence of tasks undertaken by actors within a single organizational setting. In this article, the mental health system should be understood as “comprising all the organizations, institutions and resources that are devoted to produce [mental] health actions”, and is therefore not limited to any specific sector of the economy (56).

Table 3 links clinical-administrative functions with corresponding processes and provides examples of strategies that may target each process. We grouped actions targeting clinical-administrative processes under general strategies that emerged as categories from the focused coding phase of data analysis.

**Table 3:** Examples of strategies targeting clinical-administrative processes and functions.

Functions	Processes	Examples of strategies
1. Detection function	1.1. Self-detection	Improve mental health literacy
		Provide opportunities to get reliable information
	1.2. First contact	Provide a variety of first contact opportunities
		Facilitate first contact
		Streamline first contact
	1.3. Screening	Increase awareness of screening opportunities
		Standardize screening procedures
2. Evaluation function	2.1. Services-oriented evaluation	Standardize services-oriented evaluation procedures
		Centralize patient information
	2.2. Condition-oriented evaluation	Improve evaluation skills and capabilities
	2.3. Specialized evaluation	Involve evaluation specialists early
3. Preparation function	3.1. Acceptability improvement	Destigmatize
		Improve the patient experience
		Change patients’ attitudes toward CBTs
	3.2. Options consideration	Engage in shared decision-making
	3.3. Pre-intervention	Provide information and increase skills
		Provide reassurance and support
4. Referral function	4.1. Self-referral	Emphasize affordable CBT modalities
		Facilitate contact with CBT providers
		Certify the quality of specific services
	4.2. Direct referral	Use prescriptive procedures
		Broker formal and informal collaborations
		Disseminate knowledge about locally available CBTs
	4.3. Navigation	Provide care navigation services

### 3.3 Clinical-administrative functions and processes

We identified four clinical-administrative functions. The detection function leads to the identification of the potential need for CBTs for anxiety disorders. The evaluation and the preparation functions are two distinct capabilities interacting as decision-making functions to enable intervention seeking. The referral function represents the capability to ensure provider reaching for patients actively seeking evidence-based CBTs.

#### 3.3.1 Detection function

##### 3.3.1.1 Self-detection process

Some patients may not use any mental health services unless they first identify their own potential needs through self-detection. One participant mentioned that “more than 50% of people with anxiety disorders do not consult, something must be done for them. (P19)”.

One strategy to facilitate self-detection is to improve mental health literacy in the general population, for example using information campaigns (P19), and change erroneous beliefs about anxiety disorders and their symptoms: “when you ask, “why didn't you consult?”, you know, people say “well, I thought it would go away on its own”. (P19)”.

Alternatively, participants suggested specific ways to provide opportunities to get reliable information for self-detection. “There might be information [on a state-sponsored] website to start identifying [your own potential need]. (P74)”. From a local perspective, a participant with lived experience (P36) promoted the organization of professionals-led public conferences where patients can confidentially write down questions they would not have otherwise been comfortable asking openly.

##### 3.3.1.2 First contact process

Another detection process is first contact, i.e., the voluntary encounter between a patient and an identifiable actor who may detect their potential need. First contact providers may, for example, include private sector psychologists, primary care team members, and community-based nonprofit organizations (P08, P60).

Providing a variety of first contact opportunities may be necessary for underserviced groups, including visible minorities and indigenous communities, older adults, rural communities, and veterans (P19, Doc13). Some professionals, such as clinicians involved in home care for older adults, can provide new opportunities by acting as first contact themselves (P38).

Actors currently providing first contact can also facilitate first contact by making their services easily discoverable on the internet (P51) or reachable through a single telephone number (P14). Additionally, first contact providers can mimic some nonprofit organizations to make their services more approachable: “it's less confrontational, it's less intimidating. […] You don't necessarily have to deal with a voicemail, you arrive on the spot and then you meet someone fairly quickly. (P51)”.

Finally, first contact can be improved by streamlining this process and connecting it with other structures of the mental health system. A public sector manager explained how they strived to network with school psychologists to allow them to act as first contact for students rather than isolated actors (P60).

##### 3.3.1.3 Screening process

Detection can also be fulfilled by screening, i.e., leveraging interactions with patients already receiving health services to detect a potential need for CBT independently of their awareness of this need or knowledge of CBTs' availability. In particular, the role of family physicians was highlighted by several participants as well as in Doc9: “Mental health issues are common in primary care, and the burden of identifying, diagnosing, and treating the most common disorders like anxiety and depression falls primarily on family physicians”.

Clinicians can contribute to this process by increasing their awareness of screening opportunities. A participant with peer-support experience mentioned that all clinicians who may encounter screening opportunities “should have basic training to assess that the person would need psychological support. (P06)”. To increase their awareness of screening opportunities, clinicians can seek experiences such as co-facilitating group interventions for anxiety disorders (P51, P63).

Actors can also standardize their screening procedures. A participant with policymaking experience stated that “good practice dictates that we should better detect [clinical situations such as anxiety or depression]” using tools such as the GAD-7 (P66). Indeed, GAD-7 is included in official screening-oriented recommendations to clinicians (Doc13).

#### 3.3.2 Evaluation function

Among the decision-making functions, evaluation aims to establish that CBT is needed for a given patient. Actors fulfilling the evaluation function can do so by being involved in either services-oriented evaluation, condition-oriented evaluation, or specialized evaluation.

##### 3.3.2.1 Services-oriented evaluation process

Services-oriented evaluation can be performed by actors who lack training and qualifications to diagnose anxiety disorders and may be sufficient for many patients with anxiety disorders. Indeed, participants differentiated between assessing the presence of an anxiety disorder and comparing a patient's situation with pre-established access policies. A participant with public sector management experience indicated that, in many cases, “Just with that assessment, we can offer a service (P44).”

Some participants believed that standardizing services-oriented evaluation procedures would increase the effectiveness of those evaluations to clarify whether someone would likely benefit from CBT (P44). For that purpose, Quebec's provincial mental health authorities have adapted the decision tree from the English Improving Access to Psychological Therapies (IAPT) program (Doc13).

Another strategy was to centralize patient information, enabling non-expert clinicians to leverage previous evaluations rather than relying on their skills alone. “[In the armed forces], that's how it works, it's the care navigator who […] will centralize the cases, who will receive the progress reports. So, I would really see a non-psychotherapist social worker working in this way. (P74)”. Quebec psychiatrists similarly recommend that non-specialist clinicians assessing patients' needs gather additional information from the patient's family physician and all relevant electronic medical records systems (Doc6).

##### 3.3.2.2 Condition-oriented evaluation process

In contrast with services-oriented evaluation, actors performing condition-oriented evaluation use their clinical skills to establish the relevance of CBT according to the evidence base rather than available services. This may involve physicians or psychologists diagnosing specific anxiety disorders, but other clinicians can also perform rigorous non-diagnostic condition-oriented evaluations. As described by a policymaker, clinicians such as nurses, social workers, or educators may be “seasoned professionals […] who are able to make a comprehensive assessment. […] It takes actors who can understand well, analyze situations well, maneuver with motivational issues, but who also integrate good practices. (P66)”.

Participants suggested improving clinicians' evaluation skills and capabilities. “I think there is a need for training specifically in mental health. […] If we think of a nurse clinician, yes, they will have touched on mental health, but perhaps not in a specific, specialized way. (P38)”. Mental health specialists such as psychiatrists and psychologists can improve the skills of generalist clinicians over time by providing continuing support to their colleagues (Doc6).

##### 3.3.2.3 Specialized evaluation process

Generalists vary in their interests and levels of comfort with mental health issues (P93, Doc9), and some patients will still require specialized evaluation to establish the pertinence of CBT. Therefore, some participants highlighted the benefits of involving evaluation specialists early, whether they are psychiatrists, psychologists, specialized nurses, or other professionals with mental health experience (P38, P41). Barriers to early specialist involvement may have detrimental effects as it “help[s] people to arrive much more promptly at a treatment that is likely to be effective quickly, and [their symptoms] will [be less likely to] become chronic along the way. (P41)”. As indicated by a social worker with public sector management experience: “Yes, if the resources were there, I think we would do more [specialized assessments for mental disorders]. (P44)”. Specialists may be shared between geographic areas when human resources are lacking, especially leveraging videoconference technology (P85).

#### 3.3.3 Preparation function

The second decision-making function, preparation, generates readiness to seek and engage in CBT. We found three processes that may fulfill this process: acceptability improvement, options consideration, and pre-intervention.

##### 3.3.3.1 Acceptability improvement process

Patients must find CBT acceptable if they are to engage in intervention seeking, highlighting the relevance of acceptability improvement when applicable. Depending on the situation, some degree of hesitation to seek treatment for anxiety disorders may be reasonable: “there are [workplaces where] if you reveal that you have a mental health issue to your colleagues, you're going to be fired the next week. (P51)”. Acceptability concerns may also be specific to some CBT modalities, such as group interventions that people often hesitate to consider as a first option (P44).

At the general population level, destigmatization can be attempted through awareness campaigns in social and traditional media (P51). People who have undergone CBT can also help destigmatize mainstream culture by sharing their experiences: “You have someone on the radio who says: “[…] I can tell you that psychotherapy makes a difference”. […] This is how, quietly, we will witness a culture change. (P34)”.

At the mental health services level, improving the patient experience may contribute to acceptability. If patients feel like they are facing prejudice when asking for help, they may conclude that they are better off dealing with their symptoms on their own (P06). Similarly, patients may reconsider seeking CBT if they undergo multiple evaluations and repeatedly tell their personal stories without being offered a concrete solution to their problems (P74, Doc17).

At the individual level, patients’ opinions about seeking CBT can be influenced by professionals they trust. According to a clinical psychologist, “I know that physicians work hard to get our people to consult. (P14)”. A CBT provider mentioned that referring patients to a specific professional rather than recommending CBT in abstract terms may help make even significant geographic distances acceptable (P74).

##### 3.3.3.2 Options consideration process

Patients who find CBTs broadly acceptable can engage in options consideration to finalize their decision to seek treatment. As patients weigh differently criteria such as waiting times, geographic distance, group or individual settings, and in-person or videoconference meetings (P06, P38, P51), they should be aware of the whole range of available interventions that could meet their needs. Resources such as web-based registries can help patients find this information, but even then, “you can't know what you don't know in life”; hence the frequent necessity of clinician assistance for options consideration (P38).

Any actions promoting engagement in shared decision-making can facilitate options consideration. Quebec's provincial guidelines describe some of the content of exchanges conducive to shared decision-making, including “the nature, content, and duration of any recommended treatment. (Doc13)”. A psychiatrist with policymaking experience highlighted that guidelines and similar documents could also target the general public, allowing patients to influence clinicians into considering evidence-based interventions they might be less familiar with (P82). Participants with lived experience suggested sharing information about treatment options in a written medium, allowing patients to ponder their decision outside the sometimes anxiogenic context of a mental health meeting (P06, P36).

##### 3.3.3.3 Pre-intervention process

Some patients face additional barriers to intervention seeking, such as motivational issues, for which pre-intervention may be required: “Engaging in psychotherapy is difficult, […] I believe that there is preparation to be done. (P51)”.

Actors may provide information and increase patients' skills in preparation for seeking and engaging in CBT. One participant believed that self-management workshops resulted in patients being better prepared for more intensive CBT modalities (P51). Similarly, a group CBT therapist indicated requiring every patient to engage with educational material before being eligible for targeted CBTs (Doc5).

Another angle for pre-intervention was to provide reassurance and support throughout the potentially lengthy process of CBT seeking. As clinical follow-up may encourage patients to persevere until they reach the treatment they need, clinicians should “quickly try to initiate follow-up […] rather than just directing people on a waiting list. […] You know, there is a momentum to come and get help. (P44)”. Participants suggested group interventions involving peer support as particularly beneficial in validating the experience of hardships often associated with intervention seeking (P27, P51).

#### 3.3.4 Referral function

##### 3.3.4.1 Self-referral process

Patients seeking CBT can reach a treatment provider through self-referral, with efficiency benefits for the mental health system compared to depending on a physician's prescription (P06, P93). Accordingly, one of Quebec's official guidelines' principles is to allow “self-direction of the person with a mental disorder (access directly to the services without a referral from a professional). (Doc13)”. Strategies facilitating self-referral target specific barriers to provider reaching.

Regarding financial barriers to access, actors can emphasize affordable CBT modalities and ensure that patients know whether their insurance provider covers specific CBTs or which lower-cost alternatives may be available in the nonprofit sector (P85). Patients can also be acquainted with sliding scale billing practices that may improve affordability when they self-refer despite being unable to afford a CBT providers' usual fees (P93).

Lack of information about the proper referral channels may be targeted with actions facilitating contact with CBT providers. Clinicians can inform patients about specific CBT providers they can contact (P14) and self-help resources and books that they can procure themselves (Doc5). Several participants also mentioned Quebec's *Ordre des Psychologues*’ directory of licensed psychotherapists as a valuable resource to self-refer to a local CBT provider (P34, P38, P74, Doc15).

Another barrier to self-referral is not knowing which providers or services would lead to receiving evidence-based CBT if patients were to contact them. To counteract this problem, actors can certify the quality of specific services. A psychologist with research experience clarified, for example: “Bibliotherapy is not “read any book then come back next week,” it is self-treatment guides based on the principles of CBT; there are several, so which ones will we recommend? (P08)”.

##### 3.3.4.2 Direct referral process

Some patients would prefer other options than self-referral: “if I decide that I want psychotherapy, […] I would be uncomfortable shopping for that on the internet. I would like to be referred. (P19)”. Beyond individual preferences, some patients face insurmountable barriers to self-referral: “The person goes home… either, doesn't have access to the internet, to resources, is isolated, doesn't understand the language… (P38)”. Actors involved in demand management should therefore consider direct referral to a CBT provider: “[general practitioners] should be able to directly refer users to specialized services, no matter where the user is from. (Doc17)”.

Direct referral may involve using prescriptive procedures, i.e., agreements under which a professional's conclusion that CBT is warranted is a sufficient condition to be eligible to receive that treatment (P74, Doc20). For example, insurance companies may cover CBT for anxiety disorders when recommended by a medical doctor (P34).

Brokering formal and informal collaborations may facilitate direct referral. Indeed, actors can sometimes bypass structural barriers to access by collaborating within and between clinical teams (P19). As clarified by several participants, clinicians already use both formal and informal collaborations to facilitate access (P74, P85, P93, Doc2). If practical guidelines were to recommend specific communication mechanisms, actors may be further empowered to collaborate for patients' benefit when providing demand management-related services (P93).

Direct referral can also be facilitated by disseminating knowledge about locally available CBTs and how to access them. Actors involved with clinical teams providing demand management can ensure that new clinicians have and use this knowledge (P85), which may also be disseminated to referring clinicians outside their teams through occasional meetings and post-referral feedback (P41, P44, P85). For that purpose, public sector actors are encouraged to elaborate and enact communication plans targeting patients, communities, and actors of other sectors of the mental health system (Doc13).

##### 3.3.4.3 Navigation process

In some contexts, complex supply-side structures may leave little room for either self-referral or direct referral. CBT providers also may or may not adhere to a patient's insurance plan, complicating fulfillment of the referral function (P74). Additionally, patients who attempt to self-refer without proper guidance risk engaging in interventions that may be neither evidence-based nor appropriate for their condition (P38). Therefore, navigation, i.e., aid in negotiating structurally imposed access mechanisms to reach a CBT provider, sometimes appears to be the only credible referral option.

In those cases, actors can provide care navigation services. Depending on the context, this may include public sector care coordinators or navigation specialists (Doc6, Doc13, P44, P74), insurance case managers (P38), or employer-provided navigators (P74). Our results support that actors from various disciplines and settings can and often do provide care navigation services when they estimate that patients would benefit from them.

## 4 Discussion

In this article, we proposed a model resulting from the rigorous application of constructivist grounded theory methodology and described clinical-administrative processes that actors involved in demand management may target to improve access to CBTs for adults with anxiety disorders in the community. Access to care is a fundamental tenet of the patient- or person-centeredness of care (57) and an essential dimension of healthcare quality (58). Santana et al. argued that “shifting to person-centered care requires services and roles to be redesigned and restructured” to fit this perspective better (57). Our model is a step in this direction insofar as it showcases how the roles of various actors gravitate around individual patients' access paths through clinical-administrative functions and processes.

Considering the demand management perspective, we articulated how actors who may or may not be CBT providers can leverage their role along a patient's access path with various actions and strategies. To identify which of those actions are appropriate, actors may consider how their role and context determine the clinical-administrative processes they can target to help patients progress to the next step of their access path to CBTs. Indeed, prioritizing clinical-administrative processes and identifying the strategies most likely to improve them would require a thoroughly contextualized analysis of the mental health system. Actors at the local or regional level may also find our model useful as an analytical framework to assess the person-centeredness of current services. With the involvement of clinicians and other actors involved in demand management, and most importantly, with enough input from service users with lived experience of anxiety disorders, services analysis using our model would provide some of the information needed to ensure that any redesign or restructuring accounts for the access-related challenges that patients actually face and overcome. Utilizing our model in this fashion would enable the leveraging, within demand management strategies, of continually improving access-oriented CBT delivery modalities, notably using technologies to bypass physical, financial, or sigma-related barriers (20).

Levesque et al. have previously distinguished the supply- and demand-side challenges of access, approaching demand as a characteristic of the population and supply as relating to service providers (21). A similar perspective is found in implementation science literature, whereas all “teams, individuals, and systems that adopt evidence-based interventions into practice” are considered delivery system actors (22, 59). Neither of those perspectives accounts for actors involved in demand management's specific role in providing access to evidence-based interventions. A scoping review on strategies to improve access to CBT for anxiety disorders highlighted demand-side strategies such as education campaigns and direct-to-consumer marketing (20), but it is unclear how those strategies interact with patients' access paths. A qualitative literature review summarized anxious patients' and primary care providers' perspectives on access to treatment, without integrating this information into an access-oriented framework (60). Compared to previously published research, our model is innovative in providing a sector-neutral framework of the functions actors fulfill when they integrate contextual considerations and scientific evidence to facilitate access to CBTs for patients with anxiety disorders who need those treatments.

Our model constitutes a substantive theory (26) focusing on practical considerations affecting actors involved in a mental health system comparable to the study's context. However, its substantive character does not limit our model's potential transferability to conditions other than anxiety disorders, interventions other than CBTs, or settings other than Quebec. Indeed, our model converged with what Gillam & Pencheon (23) indicated as a target in the field of demand management, i.e., “demand at all points along the path from first contact to possible referral”. We expect that a similarly structured model could have emerged from a grounded theory study using data from other contexts where demand management is warranted, especially for common mental disorders for which evidence-based psychological therapies are available; this hypothesis should be tested in the future.

### 4.1 Limitations

Some limitations of this article should be highlighted. First, number of participants in grounded theory studies is decided based on theoretical saturation, which is challenging to operationalize. While we initially expected to recruit between 20 and 25 participants, we estimate that we were able to initiate theoretical sampling and reach a sufficient level of theoretical saturation relatively early for three main reasons: the depth of data analysis conducted between interviews, the early theoretical focus of feedback provided to the interviewer by co-researchers, and the rigorous treatment of documents as additional sources of data.

Second, while conducting this study in accordance with rigorous standards for qualitative research and grounded theory, we made some decisions that may differ from what other researchers would have opted for in a similar study. We initially contemplated requesting participants' feedback on our model, but it became clear as the study progressed that participants' heterogeneity would significantly complicate this endeavor. Moreover, the value of checking back with participants may not contribute to the rigor of a grounded theory study (61). Although we documented when participants shared a lived experience of anxiety disorders, we did not specifically target this population for recruitment. We believe that this decision was sound in the context of this study focusing on demand management; future studies will be required to explore patients' perspectives. We also recruited only one participant younger than 40, potentially missing information about clinical-administrative processes with which younger clinicians would have been more familiar.

Third, since the first author conducted all interviews, participants' perceptions may have affected what they decided to share. Participants were aware that the interviewer was a psychiatrist, which may have led to self-inhibition when discussing opinions that participants perceived to deviate from the mainstream. Interview techniques were used to mitigate this limitation, such as mentioning that there were neither right nor wrong answers and recalls about the confidentiality of the interview and the priority given to the participant's point of view. The interviewer's clinical experience facilitated the creation of meaningful relationships with participants for data collection, and early feedback from co-researchers helped ensure that interviews remained focused on the research objective.

## 5 Conclusions

In this article, we presented a model of the clinical-administrative processes on patients' access paths that may be targeted by actions aiming to improve access to CBTs for anxiety disorders. We described those processes and how they connect with strategies that actors involved in demand management could implement, and we reported the grounded theory methodology that allowed us to generate those results. We believe that such a model is promising to guide access improvement by providing an integrative framework to leverage the competency of various actors at the local and regional levels. Our model should be tested with data gathered in other jurisdictions to establish its degree of transferability, and future efforts to improve access to evidence-based psychological treatments should use the resulting advancements to meet mental health needs in the general population. When a validated version of our model will be available, further research targets will include prioritizing strategies based on considerations such as effectiveness, feasibility, and acceptability. The resulting recommendations will represent an exciting prospect for advancing demand management as a field for improving access to evidence-based psychological treatments at the local level.

## Data Availability

The datasets presented in this article are not readily available because of confidentiality issues (qualitative data from interviews). Requests to access data should be directed to jean-daniel.carrier@usherbrooke.ca.
