# Supplementary material for: Demand management processes to improve access to cognitive-behavioral therapies for anxiety disorders: a grounded theory study
Source: Front Health Serv. 2024 Jan 11;3:1266987. doi: 10.3389/frhs.2023.1266987 (PMC10808741; doi:10.3389/frhs.2023.1266987)
Supplement: Supplementary file 1 [file Datasheet1.docx]

Supplementary Material

Clinical-administrative processes on patients' access paths: a grounded theory study on demand management to improve access to cognitive-behavioral therapies for anxiety disorders

Jean-Daniel Carrier^1,2*^, Frances Gallagher^3,4^, Alain Vanasse^1,4^, Pasquale Roberge^1,2,4^

^1^PRIMUS research group, Department of family medicine and emergency medicine, Université de Sherbrooke, Sherbrooke, QC, Canada

^2^Department of psychiatry, Université de Sherbrooke, Sherbrooke, QC, Canada

^3^School of nursing, Université de Sherbrooke, Sherbrooke, QC, Canada

^4^Centre de recherche du CHUS, Sherbrooke, QC, Canada

*** Correspondence:**Jean-Daniel Carrier, MD, PhD
jean-daniel.carrier@usherbrooke.ca

**Appendix 1.** Content of the interview guide (synthesis for the whole study)

**Part 1. Background**

1.1. How old are you (decades)?

1.2. What is your current occupation/profession?

1.3. What degrees/training did you get?

1.4. How many years of work experience do you have?

1.5. Can you tell me about your expertise or experience with mental health?

1.6. What organizations are you involved in that might have a connection to access to psychotherapy for anxiety disorders?

**Part 2. Access to cognitive-behavioral therapy (CBT) for anxiety disorders**

2.1. In a few words, could you give me your opinion on the current situation of access to CBT for anxiety disorders?

- Fairness
- Financial aspects
- Political issues
- Consequences

2.2. How can someone currently have access to psychotherapy/CBT?

- Process, procedures to gain access, steps, from whom, etc.
- Availability of information

2.3. In your opinion, what influences access to psychotherapy/CBT?

- Patient characteristics
- Treatment types/modalities
- Services' organization
- Professionals' role
- Available resources
- Geography

2.4. Considering what we have just discussed, what factors do you believe to be the most important regarding access to CBT for anxiety disorders in Quebec?

**Part 3. Improving access**

3.1. In an ideal world, what would facilitate access to CBT for people who could benefit from CBT for an anxiety disorder?

- Opportunities to be referred to CBT
- CBT access management mechanisms
- Procedures for identifying CBT needs
- Knowledge of services and how to access them
- Comparison with the current situation

3.2. In your opinion, what are the most promising approaches to improving access to CBT for anxiety disorders?

- Collaboration between professionals
- Organizations' role
- First/second/third lines of services
- Intersectorality
- Stepped care
- Group interventions
- Transdiagnostic approach
- Use of technology
- Self-care or self-management
- Researchers' role

3.3. What are the elements of the process of access to CBT for anxiety disorders that should be improved in Quebec? How?

- Financial aspects
- Waiting lists
- Repeated assessments
- Communication and co-ordination
- Quality improvement at the regional level

**Part 4. Follow-up questions**

4.1. Are there any other points you would like to share with us that were not covered during the interview?

4.2. Do you have any suggestions for the continuation of this research project?

- Conduct of the interview
- Themes to cover
- People to interview
- References to read
